# Anti-Idiotypic Antibodies Specific to prM Monoantibody Prevent Antibody Dependent Enhancement of Dengue Virus Infection

**DOI:** 10.3389/fcimb.2017.00157

**Published:** 2017-05-09

**Authors:** Miao Wang, Fan Yang, Dana Huang, Yalan Huang, Xiaomin Zhang, Chao Wang, Shaohua Zhang, Renli Zhang

**Affiliations:** ^1^College of Life Science and Oceanography, Shenzhen UniversityShenzhen, China; ^2^Shenzhen Center for Disease Control and PreventionShenzhen, China

**Keywords:** dengue virus, prM antibody, antibody-dependent enhancement, anti-idiotypic antibodies, *in vitro and in vivo*

## Abstract

Dengue virus (DENV) co-circulates as four serotypes (DENV1-4). Primary infection only leads to self-limited dengue fever. But secondary infection with another serotype carries a higher risk of increased disease severity, causing life-threatening dengue hemorrhagic fever/dengue shock syndrome (DHF/DSS). Serotype cross-reactive antibodies facilitate DENV infection in Fc-receptor-bearing cells by promoting virus entry via Fcγ receptors (FcγR), a process known as antibody dependent enhancement (ADE). Most studies suggested that enhancing antibodies were mainly specific to the structural premembrane protein (prM) of DENV. However, there is still no effective drugs or vaccines to prevent ADE. In this study, we firstly confirmed that both DENV-2 infected human sera (anti-DENV-2) and DENV-2 prM monoclonal antibody (prM mAb) could significantly enhance DENV-1 infection in K562 cells. Then we developed anti-idiotypic antibodies (prM-AIDs) specific to prM mAb by immunizing of Balb/c mice. Results showed that these polyclonal antibodies can dramatically reduce ADE phenomenon of DENV-1 infection in K562 cells. To further confirm the anti-ADE effect of prM-AIDs *in vivo*, interferon-α and γ receptor-deficient mice (AG6) were used as the mouse model for DENV infection. We found that administration of DENV-2 prM mAb indeed caused a higher DENV-1 titer as well as interleukin-10 (IL-10) and alaninea minotransferase (ALT) in mice infected with DENV-1, similar to clinical ADE symptoms. But when we supplemented prM-AIDs to DENV-1 challenged AG6 mice, the viral titer, IL-10 and ALT were obviously decreased to the negative control level. Of note, the number of platelets in peripheral blood of prM-AIDs group were significantly increased at day 3 post infection with DENV-1 compared that of prM-mAb group. These results confirmed that our prM-AIDs could prevent ADE not only *in vitro* but also *in vivo*, suggested that anti-idiotypic antibodies might be a new choice to be considered to treat DENV infection.

## Introduction

Dengue virus (DENV) is a mosquito-borne virus circulating with four distinct, but closely related serotypes (DENV1-4). The virus is transmitted to humans through bites of infected *Aedes albopictus* or *Aedes aegypti*. The incidence of dengue has grown dramatically around the world in recent decades. One estimate indicates 390 million dengue infections per year, of which 96 million clinically apparent cases and 3.9 billion people are at risk of infection in 128 countries (Brady et al., 2012; Bhatt et al., 2013).

Most people who are infected with DENV only show symptoms of mild dengue fever, but some may progress to severe dengue hemorrhagic fever (DHF) or dengue shock syndrome (DSS). Infection by one serotype provides lifelong immunity against that particular serotype (Reich et al., 2013; Forshey et al., 2016). However, subsequent infection by another serotype may increase the risk of developing severe dengue (Sangkawibha et al., 1984; Guzman et al., 2000; Screaton et al., 2015). It has been proven that infants born to dengue immunized mothers have higher risk of DHF during primary infection with DENV (Chau et al., 2008; Clapham et al., 2015). One explanation of severe DENV infections is the theory of antibody dependent enhancement (ADE) raised by Halstead in 1977 (Halstead and O'Rourke, 1977). The enhancing antibodies facilitate virus entry into susceptible myeloid cell types via FcR pathway and trigger the massive release of inflammatory and vasoactive mediators, which contribute to the disease severity (Halstead and O'Rourke, 1977; Flipse et al., 2013). High viral load, liver injury and vascular leakage are the cardinal features of severe dengue (Wichmann et al., 2004). Both sera interleukin 10 (IL-10) and alanine aminotransferase (ALT) levels were higher in those with severe dengue compared to those with mild dengue (Malavige et al., 2013; Liao et al., 2015). And high levels of IL-10 and ALT were found to associate with liver failure in dengue infections (Ferreira et al., 2015; Fernando et al., 2016). Therefore, IL-10 and ALT can be taken as the biomarkers of sever dengue disease (John et al., 2015).

Dengue virus (DENV) contains 180 copies of envelop (E) and membrane (M) protein. The premembrane (prM) protein, which consists of two moieties of the pr and M domins, is the precursor of M protein. During virus maturation, furin protein cleaves prM protein to M protein in the trans-Golgi compartment. However, about 30% immature virions are released from infected cells (Zybert et al., 2008). A study reported that prM antibodies of DENV-2 facilitate efficient binding and cell entry of the immature type 2 viral particles into Fc-receptor-expressing cells (Rodenhuis-Zybert et al., 2010). The immune response in human to DENV is mainly caused by prM antibodies, which are highly cross-reactive among the four DENV serotypes (Beltramello et al., 2010; Dejnirattisai et al., 2010; Flipse and Smit, 2015). They do not neutralize infection but potently promote ADE even at high concentrations (Beltramello et al., 2010; Dejnirattisai et al., 2010). Therefore, it is the major challenge to design effective vaccine without triggering ADE activities because most current vaccines containing naive dengue prM protein (Ramakrishnan et al., 2015).

The lack of suitable animal models hinders the development of anti-dengue drugs. Currently, there is no specific treatment for severe dengue disease. It is urgent to search novel therapeutic strategies to block ADE. Anti-idiotypic antibodies (Anti-ids) are antibodies against the antigen binding sites of another antibody. Anti-ids have been studied and used in a variety of situations including therapeutic agents (Pan et al., 1995). Thus, we firstly developed mouse polyclonal anti-ids (prM-AIDs) targeting the antigen binding sites of DENV-2 prM monoclonal antibody (prM mAb). Then we displayed the anti-ADE effects of the prM-AIDs in both K562 cells and a recently reported IFN-α and -γ receptors deficient mouse model (C57BL/6 strain, AG6 strain) for DENV infection (Liu et al., 2016). Our results suggested that anti-ids specific to prM antibody might be considered as a potential candidate to reduce ADE in DENV infection.

## Result

### ADE of DENV infection mediated by human anti-denv-2 sera in K562 cells

**Figure 1:**
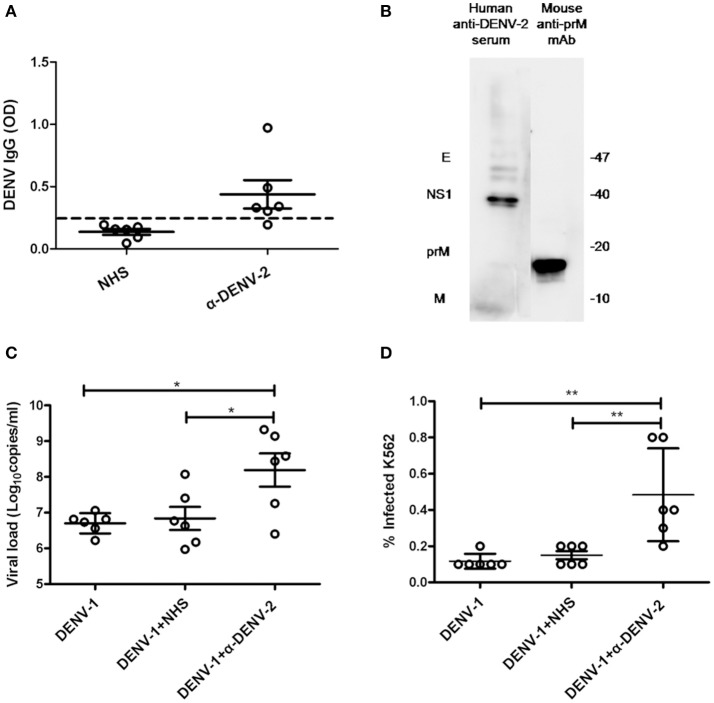
**Cross-reactivity and enhancing properties of human anti-DENV-2 sera. (A)** The OD of DENV IgG in the naive human sera (NHS) and human anti-DENV-2 sera (α-DENV-2) was determined by ELISA (*n* = 6). **(B)** Western blot of DENV-1 showing reactivity with antibodies of dengue E, NS1, prM and M in human anti-DENV-2 sera. **(C)** Human anti-DENV-2 sera was incubated with DENV-1 for 1 h at 25°C, then they were transferred to K562 cells at MOI of 1 and kept incubation for 72 h. Viral RNA copies of infected supernatant were quantified by qRT-PCR. **(D)** Infected cells were determined by flow cytometry. Error bars show the means ± SEM and pairwise comparisons were performed by unpaired test (^*^p < 0.05, ^**^p < 0.01). Dotted line presents the limit value to be considered positive.

To confirm whether anti-DENV-2 antibodies could induce ADE to DENV-1 infection, we collected six DENV-2 infected human sera (α-DENV-2). Dengue IgG in the sera was detected by ELISA. Results showed five of them were considered positive of dengue IgG. The average optical density values (O.D.) of DENV IgG in α-DENV-2 and control group were 0.439 ± 0.278 and 0.137 ± 0.056, respectively (Figure 1A). To confirm whether proteins of DENV-1 cross-reactive with human anti-DENV-2 sera and a commercial mouse DENV-2 prM mAb, we used DENV-1 as antigens and the mixed sera of six patients or prM mAb as the first antibody for western blot. Results showed human anti-DENV-2 sera contained corresponding anbodies for prM, M, NS1 and E protein of DENV-1. Anti-DENV-2 prM mAb could react with DENV-1 prM protein (Figure 1B).

Dengue virus (DENV) mainly infected monocytes, macrophages and dendritic cells in patients (Jessie et al., 2004). Human monocyte cell line K562 (Fc receptor-bearing) was a common cell type to demonstrate the ADE of DENV *in vitro*. To confirm whether the human anti-DENV-2 sera could induce ADE, we infected K562 cells with DENV-1, then determined the viral load in the supernatant by qRT-PCR and detected infected cell number by flow cytometry. PCR results showed 10–100 fold enhancement of infection in four human anti-DENV-2 sera (Figure 1C). Similarly, anti-DENV-2 sera also led to a significant increase of infected cells (Figure 1D).

### ADE of DENV infection mediated by prM mAb in K562 cells

**Figure 2:**
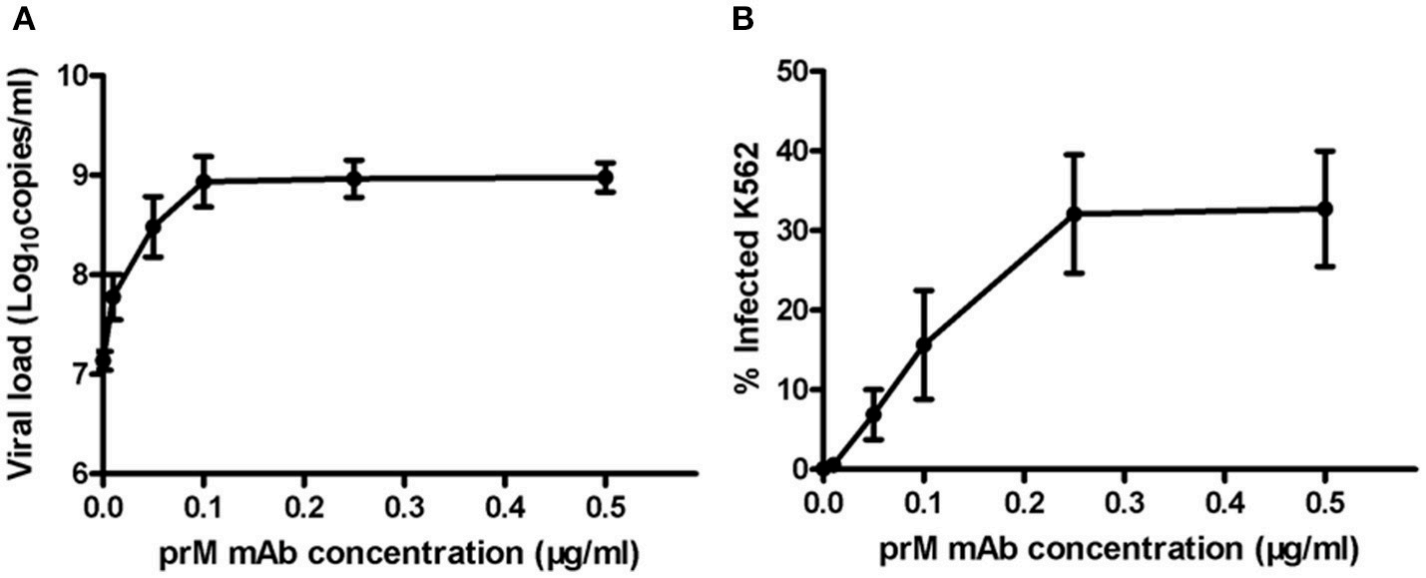
**ADE of DENV replication in K562 cells mediated by prM mAb**. prM mAb was diluted to a series of different concentrations, then they were incubated with DENV-1 for 1 h at 25°C and transferred to K562 cells at MOI of 1. Infected cells were incubated for 72 h at 37°C. **(A)** Viral RNA copies in infected supernatant were quantified by qRT-PCR. **(B)** Infected cells were determined by flow cytometry. Data are expressed as means of three independent experiments. Error bars show the means ± SEM.

Next, to determine ADE effect of mouse prM mAb, we incubated K562 cells with DENV-1 (MOI = 1) and a series of diluted prM mAb for 72 h. Results showed that prM mAb had strong enhancement effect for DENV-1 infection in a concentration-dependent manner. A 100-fold enhancement of DENV replication was detected with 0.1, 0.25 and 0.5 μg/ml prM mAb when analyzed by qRT-PCR (Figure 2A). Less than one percent of monocytes were infected with DENV-1 without prM mAb. However, the percent of infected cells displayed an increased trend along with higher prM mAb concentration. The largest DENV-1 infected cell percentage was detected when the added prM mAb concentration was increased to 0.25 μg/mL(Figure 2B). These results illustrated that prM mAb could enhance the infection of heterotypic virus and might played a vital role in dengue disease.

### Characterization of prM-AIDs

**Figure 3:**
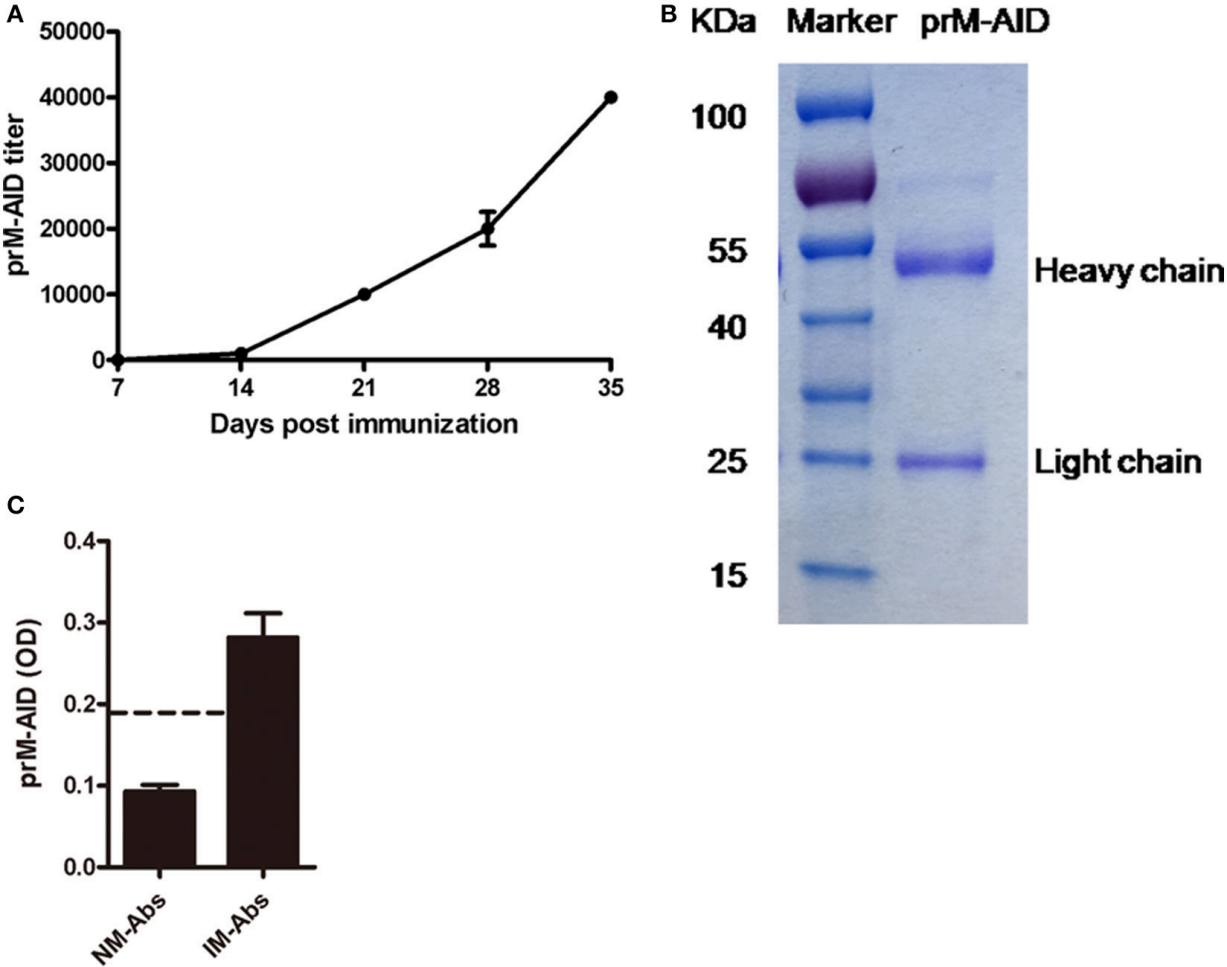
**Characterization of prM-AIDs. (A)** The titer of prM-AIDs in immunizzed sera were determined by ELISA every 2 weeks (*n* = 6). Dilution of immunized or naive mouse sera were coated in 96-well plates. Biotinylated prM mAb were added. Bound prM mAb was detected by addition of streptavidin-HRP. **(B)** Purified prM-AIDs was identified by SDS-PAGE. **(C)** The specificity of prM-AIDs was analyzed by ELISA. Diluted naive mouse antibodies (NM-Abs), or immunized mouse antibodies (IM-Abs) were coated in 96-well plates. Biotinylated prM mAb were added. Bound prM mAb was detected by addition of streptavidin-HRP. Dotted line shows samples were considered positive if Positive/Native ration>2.1.

Then, to prevent ADE induced by prM mAb, we developed anti-idiotypic antibodies (prM-AIDs) specific to prM mAb by immunizing balb/c mice. prM-AIDs in immunized mice were detected by ELISA and the titer was increased continually from day 7 after mouse immunization (Figure 3A). When the titer of anti-sera reached 1:40000 at day 35 (Figure 3A), mice were sacrificed to collect blood. prM-AIDs in the sera and naive mouse antibodies (NM-Abs) were purified by Protein-G column. The quantity and quality of purified prM-AIDs was determined by SDS-PAGE and ELISA. Results showed the purified antibody's heavy chain (about 55KDa) and light chain (about 25KDa) (Figure 3B). The optical density values of prM-AIDs in the naive mouse antibodies (NM-Abs) or immunized mouse antibodies (IM-Abs) and 0.093 ± 0.014 and 0.28 ± 0.073, respectively (Figure 3C). These data demonstrated balb/c mice immunized with prM mAb produced a high level of prM-AIDs.

### prM-AIDs inhibited ADE of DENV infection in K562 cells

**Figure 4:**
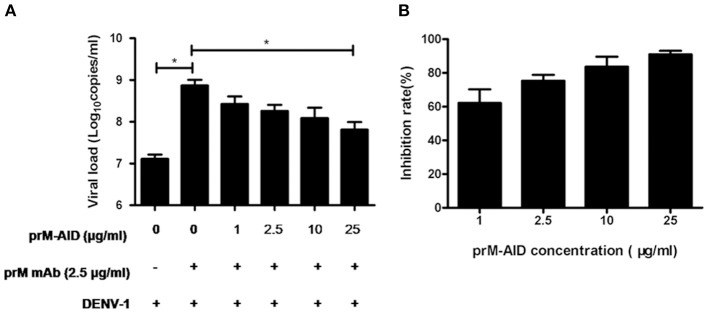
**ADE of DENV infection blocked by prM-AIDs in K562 cells. (A,B)** prM-AIDs was incubated for 1 h at 25°C with DENV-1 and 0.25 μg prM mAb at an MOI of 1. The mixtures infected K562 cell for 72 h. The virus in the supernatant was determined by qRT-PCR. Data are expressed as means of three independent experiments. Error bars show the means ± SEM and paired comparisons were performed by unpaired test (^*^p < 0.05).

To determine whether our purified prM-AIDs could inhibit ADE of DENV infection, K562 cells were infected with DENV-1 virus and prM antibody and various concentrations of prM-AIDs. prM mAb was added instead of prM-AIDs as a control group. The results showed that if only adding prM mAb and DENV-1, viral load in supernatant was significantly higher than the DENV-1 alone group. But when we added prM-AIDs, the viral load was decreased in a dose-dependent manner. Compared with the prM mAb group (8.2 ± 2.8 × 10^8^ copies/mL), the viral load in the supernatant with prM-AIDs (25 μg/mL) was dramatically decreased to 7.8 ± 3.4 × 10^7^ copies/mL (Figure 4A). Therefore, prM-AIDs significantly diminished ADE induced by prM mAb through decreasing 90% viral replication (Figure 4B).

### ADE of DENV infection mediated by prM mAb in AG6 mice

**Figure 5:**
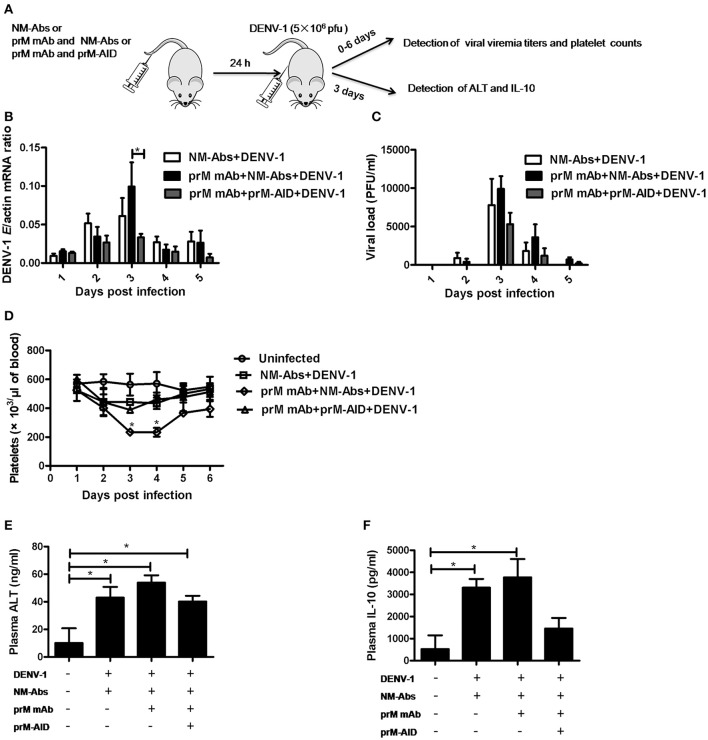
**prM-AIDs inhibited ADE of DENV infection ***in vivo***. (A)** Schematic representation of the study design. Mice were first administered with naive mouse antibodies (NM-Abs), prM mAb and prM-AIDs, then 24 h later they were challenged with DENV-1 by *i.p*.. Blood was collected from the vail veins from 0 to 5 days post-infection. **(B)** Viral copies in the blood cells were quantified by qRT-PCR. **(C)** Infectious viral particles in the plasma were assessed by plaque assay. **(D)** Platelets in blood were counted using a hemocytometer under a light microscope. **(E,F)** The levels of ALT and IL-10 in plasma were measured by ELISA at day 3 post-infection. *n* = 4–5 mice per group. Error bars show the means ± SEM and pairwise comparisons were performed by non-parametric Mann-Whitney test (^*^p < 0.05).

To assess the ability of prM-AIDs to block ADE *in vivo*, we utilized a recently described AG6 mouse model (interferon-α and γ receptor deficient mice) for ADE of dengue infection. (Figure 5A) prM mAb with or without prM-AIDs were transferred into AG6 mice by i.v. prior to infection with DENV-1. Antibodies from naive mouse sera were purified as control antibodies (NM-Abs). Viral RNA load in blood cells was detected by qRT-PCR (Figure 5B), while infective viral particles in plasma was detected by plaque assay (Figure 5C). QA peak viremia titer in blood cells was detected at day 3 post infection (Figure 5B). Compared with the NM-Abs group, the viremia titer was 1-fold higher in the prM mAb group (Figure 5B). At day 3 after inoculation, the platelet counts in DENV-1 injected mice were significantly lower than that of uninfected mice. Challenging with prM mAbs significantly reduced platelets (Figure 5D).

To further explore the inflammation response and liver damage of ADE infection, we also detected ALT (Figure 5E) and IL-10 (Figure 5F) in the plasma of mice at days 3 post infection by ELISA assay. Compared with the uninfected groups, a significant higher level of ALT and IL-10 were observed in the DENV infected groups (Figures 5E,F). Compared to the pure DENV-1 infected mice group, the level of ALT and IL-10 were increased in the prM mAb mice group. These data show that ADE of DENV-1 infection in AG6 mice by passive transfer prM antibody.

### prM-AIDs inhibited ADE of DENV infection in AG6 mice

However, by comparison, a significant difference in viral load of blood cell between the mice that received prM-AID and the mice that received prM mAb at day 3 post infection (Figure 5B). Similarly, the viral load in the plasma also showed a peak at day 3 post infection. Although the viral titer in the prM-AIDs group displayed the lowest trend, there was no statistically significant difference among all the groups. Compared with the prM mAb group, the mean viremia titer in the prM-AIDs group decreased about 1-fold (Figure 5C).

Interestingly, the number of the platelets in the prM-AIDs challenged mice was significantly higher than that of NM-Abs group at day 3 post infection (Figure 5D). Besides, challenged with prM-AIDs showed decreased trend of the level of ALT and IL-10 than the prM mAb mice group and naive mouse antibodies group (Figures 5E,F).

## Discussion

In this study, we firstly confirmed ADE to DENV-1 infection induced by human anti-DENV-2 sera and murine anti-DENV-2 prM mAb in K562 cells. Then we developed and characterized prM-AIDs targeting the antigen-binding site of prM mAb. We showed that prM-AIDs significantly inhibited ADE mediated by prM mAb *in vitro*. Importantly, administration of prM-AIDs to AG6 mice largely reduced the disease severity of DENV-1 infection.

Secondary infection with another DENV serotype carries an increased risk of severe disease (Soo et al., 2016). A high viral load in blood, severe thrombocytopenia, and platelet dysfunction may result in increased capillary fragility (Ojha et al., 2017), clinically manifested as petechiae, easy bruising, and gastrointestinal mucosal bleeding (Deshwal et al., 2016), which is characteristic of DHF/DSS. ADE has been proposed as an underlying pathogenic mechanism of DHF/DSS (Halstead, 1970). On one hand, enhancing antibodies can bind DENV through Fab fragments, on the other hand, virus-antibodies complex still could recognize Fc receptor-bearing cells via their Fc region, leading to increased virus uptake and replication (Gan et al., 2017). Many evidences showed viral premembrane protein prM mAbs broad cross-activity with the four DENV serotypes and showed poor neutralization function but potently promote ADE (Beltramello et al., 2010; Dejnirattisai et al., 2010). To confirm the ADE induced by prM mAb, we incubated DENV-1 infected K562 cells with either human anti-DNEV2 sera or mouse prM mAb. The K562 cell line was chosen as a simple and easily interpretable model system for the *in vitro* study of ADE and ADE inhibition as it only expresses the FcγII receptor (Clark et al., 2016). ADE induced by patients' sera had once been reported in laboratory virus strains (Chaichana et al., 2014; de Alwis et al., 2014). Here, a clinical DENV-1 isolate was taken to perform ADE assay. We confirmed clinical isolate DV1 infection was enhanced by human anti-DV2 sera or murine anti-DV2 prM mAb in Fcγ receptor-bearing K562 cells. Besides, since the clinical isolated DENV-1 virus strain and the anti-DENV-2 sera were both derived from Guangdong local DENV infected patients. It indicates that people living in Guangdong who were primarily infected with DENV-2 have high risk of developing severe dengue disease once they are further attacked by DENV-1.

To date, no entirely successful attempt of active immunization or drug treatment in the field of DENV has been published. The only licensed tetravalent dengue vaccine Dengvaxia (CYD-TDV) which was developed by Sanofi Pasteur and TAK-003 (Takeda) as a candidate vaccine both showed moderate efficacy against dengue infection (Osorio et al., 2014; Godoi et al., 2017). However, considering the ADE activities, current vaccines have a potential risk of aggravating disease (Aguiar et al., 2017). A recent report published on *science* suggested that vaccination of Dengvaxia in low-transmission settings might increase the incidence of more severe “secondary-like” infection and hospitalizations (Ferguson et al., 2016). Meanwhile, some anti-viral compounds have shown efficient ADE inhibition of DENV (Ayala-Nunez et al., 2013; Flingai et al., 2015). Anti-TNFα therapy totally protected the DENV-2 infected A129 mice (Martinez Gomez et al., 2016). Importantly, humanized anti-DENV antibodies engineered with mutations in Fc region were developed to prevent binding to FcR for the treatment of dengue disease in AG129 mice (Goncalvez et al., 2007; Balsitis et al., 2010; Beltramello et al., 2010; Ramadhany et al., 2015). A recent study reported plant-produced anti-dengue virus monoclonal antibodies exhibited reduced ADE activity in FcR expressing human cells (Dent et al., 2016). However, to our knowledge, no method mentioned above has been widely accepted. As a consequence, passive immune therapy of anti-idiotypic antibodies by blocking enhancing antibodies may provide an alternative strategy for the treatment of dengue, especially for patients who have already acquired primary infection or reside in endemic areas.

When one antibody binds to the variable region of another antibody, it is referred to as an anti-idiotypic antibody. Over the past years, anti-idiotypic antibodies have been studied and used in a variety of situations including attempts to use them as therapeutic agents (Denapoli et al., 2017). They are ideal for bioanalytical assays in preclinical research and clinical drug monitoring due to their high specificity and sensitivity (Bulashev et al., 2016). One research showed that anti-idiotypic antibodies could inhibit the binding between autoantibodies and autoantigens, resulting to prevent the development of autoimmune disease (Hampe, 2012). In this study we produced high titer of prM-AIDs by immunizing mice with prM mAbs. Results confirmed that prM-AIDs significantly inhibited DENV-1 replication in K562 cells in a dose-dependent manner. Although other modified antibodies could reduce their enhancing activity (Dent et al., 2016; Wang et al., 2017), they couldn't block the enhancing antibodies that already existed in primary infection. Polyclonal antibodies can be easily raised, as their production steps do not require any sophisticated laboratory facility. Therefore, anti-idiotypic antibody therapy represents a novel approach for dengue disease prevention and treatment. They have great potential to be used in combination with viral replication inhibitors that will decrease the emergence chance of resistant virus strains.

Many ADE-related studies were limited in *in vitro* assays (Dent et al., 2016; Wang et al., 2017). The evidence demonstrating ADE *in vivo* was first described by Healstead in rhesus monkeys (Halstead, 1979). Recently, a new animal model AG6 mice (interferon-α and γ receptor deficient mice) for dengue infection was reported to study the function of secreted DENV NS1 protein (Liu et al., 2016). In this model, DENV could lead to lethal disease characterized by reduced white blood cells and platelets as well as vascular leakage (Orozco et al., 2012; Liu et al., 2016), all features associated with severe dengue symptoms in humans (Halstead, 2007). Therefore, we chose AG6 mice as an animal model system for the study of ADE and ADE inhibition *in vivo*. ADE of DENV-1 replication in AG6 mice was demonstrated by passive antibody transfer. Increased virus titers, IL-10 and ALT in the blood were observed in the mice after prM antibody injection by i.v. Besides, the number of the platelets was 2-fold lower in mice challenged with prM antibody than that in the control mice. These results were similar to those in another ADE mice model-AG129 (IFN-α and β receptor deficient) (Balsitis et al., 2010; Martinez Gomez et al., 2016). Here, we first demonstrated ADE in AG6 mice by passively transfering prM mAb, although the enhancing effect was not very significant compared with other studies using different ADE mice models. We proposed that the different virus strains as well as viral challenging routes and doses might impact the disease progression and manifestations. For example, in this study, we challenged mice with DENV-1, which was believed to be weaker than DENV-2 used in other studies in pathogenesis.

Next, to confirm whether our prM-AIDs had the same inhibitory effect *in vivo* similar to that in cells, we challenged AG6 mice with both DENV-1 and prM-AIDs. Results showed prM-AIDs were capable to block the ADE effect and reducing the viral burden to an equivalent level to the DENV-1 control group in absence of prM mAb During the ADE and severe dengue symptoms, reduced platelets, platelet dysfunction as well as liver damages were common pathogenesis mechanisms. Therefore, in this study, besides the viral load in blood cells and the plasma, the levels of IL-10, ALT and platelet counts in the blood were also detected. We found the levels of IL-10 and ALT were decreased by prM-AIDs, suggesting that mice had reduced inflammatory responses and liver damages. The number of platelets in the peripheral blood of prM-AIDs group returned to normal level, which hinted that vascular damages and bleeding possibility were also reduced. These results demonstrated that the prM-AIDs could inhibit ADE induced by prM mAb *in vivo* at least from immune responses as well as liver functions and platelet counts. The mechanisms of anti-ADE by prM-AIDs might be that they specifically bound to the antigenic determinants of prM mAbs, resulting in inhibition of the binding between prM mAbs and viruses. Further studies needed to be done to confirm the detailed anti-ADE mechanisms of prM-AIDs.

Taken together, we successfully demonstrated the ADE in both cell level and in a recently reported AG6 mice model by challenging prM mAbs. Then, we developed prM-AIDs specific to this anti-dengue prM mAb. Furthermore, the prM-AIDs successfully inhibited ADE not only *in vitro* but also protected mice avoiding severe inflammatory responses as well as liver damages *in vivo*. It provided a new strategy to develop specific treatment for severe DENV infection.

## Materials and methods

### Ethics statement

All patient samples and data were assigned institution-specific identification numbers to ensure patient anonymity.Balb/c mice were purchased from Guangdong medical Laboratory Animal Center. The mice were bred and maintained under SPF animal house in Shenzhen Center for Disease Control and Prevention. C57BL/6 mice deficient in type I and type II interferon (IFN) receptors (AG6 mice) were purchased from Institute Pasteur of Shanghai, Chinese Academy of Science, which is bred and maintained under SPF animal house in Tsinghua University. All experiments procedures were approved and performed according to the guidelines of the Experimental Animal Welfare and Ethics Committee of Shenzhen Center for Disease Control and Prevention.

### Cell lines and virus

The Aedesalbopictus cell line C6/36 and Baby Hamster Kidney-21 (BHK-21) cell line were cultured in Dulbecco's modification of Eagle's medium (DMEM). Human erythroleukaemic K562 cells were grown in Roswell Park Memorial Institute (RPMI) 1640 medium. All media were supplemented with 10% fetal bovine sera, 100 U/mL penicillin, and 100 μg/mL streptomycin. C6/36 cells were propagated at 28°C and BHK-21 and K562 cells were propagated at 37°C. All the materials used for cell culture were purchased from GIBOCO and cells were from China Center for Type Culture Collection.

DENV-1 was isolated in our laboratory from a patient during a DENV outbreak in Shenzhen, China in 2014. Virus was propagated in C6/36 cell line. After 3-4 days, the supernatant was harvested, and cell debrises were removed by centrifuge at 10000 rpm for 10 min at 4°C. DENV-1 was used after being passaged for 3 times in C6/36 cells. To increase the virus titer, the supernatant were concentrated by centrifuge at 4,000 g for 30 min at 4°C using filtration device (Millipore, Cat. No# UFC901008). Virus stocks were stored at −80°C.

### Anti-DENV-2 sera

Human anti-DENV-2 sera samples were obtained from patients during a DENV outbreak in Guangdong, China. Human anti-DENV-2 sera samples and native human sera samples were collected from Shenzhen Center for Disease Control and Prevention.

### Anti-prM monoclonal antibody

Dengue virus (DENV) type 2 prM IgG1 monoclonal antibody, directed against pre-membrane glycoprotein of DENV serotype 2 was purchased from Thermo (Cat. No#, MA1-71252). To remove sodium azide in the supernatant, the antibody was further purified by the Montage Prosep-G kit (Millipore, Cat. No# P36491) and quantitated by the BCA Protein Quantitate Kit (Thermo Scientific, Cat.No# 23225) ccording to manufacturer's instructions.

### Plaque forming assay

The titer of virus was determined by plaque assay in BHK-21 cells. BHK-21 cells were seeded in 6-well plate in DMEM with 10% FBS at 37°C. Next day, virus was serially diluted with DMEM medium and incubated with BHK cells for 1 h. Subsequently, the viral suspension was removed, and DMEM medium containing 2% FBS and 1% low-melting point agarose were added. After incubation at 37°C for 6 days, cells were fixed with 4% formaldehyde for 2 h. After the overlay was removed, cells were stained by crystal violet for a few minutes and washed carefully by water. Plaques were calculated by naked eyes.

### Cross-reactivity analysis between DENV-1 and human anti-DENV sera or prM mAb by western blot

The DENV-1 supernatant was electrophoresed through 12% SDS/polyacrylamide gels and then electrotransferred to nitrocellulose membrane (Millipore). The membrane was blocked with 5% BSA in TBST before incubation overnight with human anti-DENV sera (1:100 diluted) or prM mAb at 4°C. Wash the membrane with TBST four times. Peroxidase-Labeled Antibody to Human IgG (KPL, Cat. No# 04-10-20, 1:2,000 dilute) or Peroxidase-Labeled Antibody to mice IgG (Abcam,Cat. No#ab6789, 1:100,000 dilute) was added in TBST with 5% BSA for 2 h at room temperature. After washing the membrane, bands were detected by using SuperSignal West Pico chemiluminescent substrate (Thermo Scientific, Cat. No#HF104626).

### Analysis of ADE induced by human sera or prM mAb in K562 cell

Human sera or prM mAb was incubated with DENV-1 at an MOI of 1 for 1 h at 25°C in shaking table at 80 rpm to form virus-antibody complexes. The mixtures infected K562 cells in 24-well plates (1 × 10^5^ cells/well) at 37°C for 72 h. The number of infected cells was determined by flow cytometry. The virus in the supernatant was determined by qRT-PCR.

DENV-1 infected and mock K562 cells were harvested and washed with cold phosphate buffered saline (PBS). To block cell surface FcR, incubate cells with 0.5 μg Human BD Fc Block™(BD, Cat. No#564220) for 20 min at room temperature. Fixation, permeabilized and intracellular fluorescence labeling were performed by Fixation/Permeabilization solution (BD, Cat. No#554714) according to manufacturer's instructions. For labeling, cells were incubated with anti-dengue virus E glycoprotein antibody (abcam, Cat. No# ab41329, 1:20 dilute) and then labeled with Donkey F(ab')2 Anti-Mouse IgG H&L (Alexa Fluor® 488,abcam, Cat. No#ab181289). Finally, cells were suspended in PBS containing 1% FBS and subjected to flow cytometry.

RNA in the supernatant was extracted using Viral RNA kit (Roche, Cat. No#13438700). qRT-PCR was carried out with DENV General-type Real Time RT-PCR Kit (Shanghai ZJ, Cat. No#ER-0101-02). Amplification conditions were 45°C for 10 min, 95°C for 15 min and 40 cycles of 95°C for 15 s, 60°C for 1 min.

### Preparation of prM-AIDs

To obtain prM-AID, six female Balb/c mice (6 weeks of age) were subcutaneously immunized with dengue prM mAb three times at 2-week intervals in Freund's complete(sigma, Cat. No#F5881) or incomplete adjuvant (sigma, Cat. No#F5506). Blood samples were collected from the tail vein of immunized mice to determinate the titer of antibody. Seven days after the final immunization, mice were sacrificed to take the blood. The sera were separated from the blood by centrifuge at 2,500 g for 30 min at 4°C and the products was purified from sera using the Montage Prosep-G kit (Millipore Cat. No#P36491). The polyclonal antibodies were concentrated using centrifugal filter device (Millipore) and quantitated by the BCA Protein Quantitate Kit (Thermo, Cat. No#23225) according to manufacturer's instructions. The purity of polyclonal antibodies was identified by sodium dodecyl sulfate-poly-acrylamide gel electrophoresis (SDS-PAGE) on 12% polyacrylamide gels under denaturating and reducing conditions and evaluated by Coomassie blue staining.

### Detection of prM-AIDs by ELISA

To dectect prM-AIDs titer in the immunized sera, serially diluted control and immunized sera were coated in 96-well plates at 4°C overnight. And to identify prM-AIDs in purify antibodies, antibodies purified from naive mice as negative control, and purify anti-idiotypic antibodies from immunized mice were coated in 96-well plates at 4°C overnight. The plates were blocked by 2% BSA in PBST for 1 h at room temperature. The prM mAb was biotinylated by using NH2-reactive biotin (Elabscience, Cat. No#EBLK0002). After washing with TBST, biotinylated prM mAb was added and incubated for 1 h at 37°C. Plates were washed four times with TBST. Streptavidin-HRP (1:10,000 diluted, Beyotime, Cat. No#A0303) were added and incubated for 30 h at 37°C. Wash the plates as above. TMB was added as the substrate, and the reaction was stopped by H_2_SO_4_ stop solution. The OD at 450 nm was measured with an iMarkTM Microplate Reader. Samples were considered positive if P/N ration>2.1.

### ADE inhibition assay by prM-AID in K562 cells

prM-AIDs was incubated for 1 h at 25°C in shaking table at 80 rpm with DENV-1 and prM mAb at an MOI of 1. The mixtures infected K562 cells in 24-well plates at 37°C with 5% CO_2_ for 72 h. The virus in the supernatant was determined by qRT-PCR.

### ADE inhibition by prM-AID in AG6 mice

Twenty six to eight week-old AG6 mice were randomly into four groups. Antibodies were purified from naive mouse sera (NM-Abs) as control antibodies. Mice were injected 100 μg NM-Abs, 20 μg prM mAb and 100 μg NM-Abs or 20 μg prM mAb and 100 μg prM-AID by intravenous injection (*i.v*) into the tail vein then intraperitoneal (*i.p*) infected 24 h later with 10^6^ PFU of DENV-1. Blood samples from each mice were collected from the tail vein daily for the next 5 days. Viremia titers were detected from day 1 to day 5 post infection. Platelets were count from day 1 to day 5 post infection. The level of IL-10 and ALT were determined at day 3 post infection.

### Quantitative analysis of DENV-1 viremia in AG6 mice

10 μl whole blood was collected each day after viral challenge into tubes containing sodium citrate. Samples were centrifuged at 6,000 g and 4°C for 5 min to separate plasma and blood cells. Viral load in the plasma was determined by plaque. RNA was extracted from blood cells using an RNA Mini kit (Qiagen, Cat. No#154025157) and reverse transcripted into cDNA using an iScript cDNA kit (Bio-Rad, Cat. No#1708891). Viral genomes were quantified via TaqMan qPCR amplification. The DENV-1 primer pairs were GCTCCCACGTCGGAAATACA and TGTTCTAGGTGAGCAGTCCAATG. The probe was FAM 5′CTGACTGACTACGGAGCC-3′ TAMRA. The sequence of mouse actin primer pairs were AGCCATGTACGTAGCCATCCA and TCTCCGGAGTCCATCACAATG. The probe was FAM 5′-TGTCCCTGTATGCCTCTGGTCGTACCAC-3′ TAMRA. Gene quantities were normalized against mouse actin.

### Detection of IL-10 and ALT by ELISA

IL-10 (Abcam,Cat. No#ab46103) and ALT (Cloud-clone, Cat. No#SEA207Mu) in the infected and mock-infected mice was quantified using commercially available ELISA kits. The experiment was performed according to manufacturer's instructions.

### Statistical analysis

Unpaired *t*-test was used to comparison of viral road and percentage of infection in ADE and ADE inhibition assay. Non-parametric Mann-Whitney test were used for pairwise comparisons of viral road, IL-10, ALT and platelets in AG6 mice. All calculations were performed in GraphPad Prism 5.0 software.

## Author contributions

RZ designed the experiments; RZ, MW, XZ, and YH wrote the manuscript and performed the majority of the experiments and analyzed data; FY, DH, CW, and SZ supplied material and performed a part of the experiments. All authors reviewed, critiqued, and provided comments to the text.

## Funding

This work was financially supported by the National major science and technology research projects of China (No.2016YFC1202001) and science and technology research projects of Shenzhen (No. JCYJ20160427151920801).

### Conflict of interest statement

The authors declare that the research was conducted in the absence of any commercial or financial relationships that could be construed as a potential conflict of interest.
